# 聚多巴胺涂敷的聚酰胺-胺功能化二氧化硅复合材料用于水样中苯甲酰脲类杀虫剂的分散微固相萃取

**DOI:** 10.3724/SP.J.1123.2022.03012

**Published:** 2022-10-08

**Authors:** Xiaoyan CUI, Wenyu MA, Xiwen LIN, Runhua LU, Haixiang GAO, Wenfeng ZHOU

**Affiliations:** 中国农业大学理学院应用化学系, 北京 100193; Department of Applied Chemistry, College of Science, China Agricultural University, Beijing 100193, China

**Keywords:** 分散微固相萃取, 苯甲酰脲类杀虫剂, 树状分子, 聚酰胺-胺, 聚多巴胺, dispersed micro solid-phase extraction (D-μ-SPE), benzoylurea insecticides (BUs), dendrimer, polyamidoamine (PAMAM), polydopamine (PDA)

## Abstract

近年来,由农药残留导致的环境污染问题已引起社会的广泛关注,开发便捷高效的分析方法对农药残留进行检测和监测十分必要。研究设计并成功制备了聚多巴胺涂敷的聚酰胺-胺树状分子功能化的二氧化硅复合材料(SiO_2_-PAMAM-PDA),并采用透射电镜对其进行表征。开发了以此复合材料为吸附剂的分散微固相萃取方法(D-μ-SPE),并结合高效液相色谱对水基质中的4种苯甲酰脲类杀虫剂(BUs)残留进行了富集检测。多巴胺结构中含有丰富的苯环、氨基及羟基,可与目标物形成氢键、*π-π*相互作用,从而增强了材料对苯甲酰脲的萃取能力。对吸附剂用量、萃取时间等可能影响萃取效率的条件进行了单因素优化。在最优条件下,该方法的线性范围在10~500 μg/L之间,根据3倍信噪比(*S/N*)计算所得的检出限(LOD)为1.1~2.1 μg/L,回收率为82.8%~94.1%,相对标准偏差(RSD)为2.1%~8.0%。将建立的方法与已报道的以苯甲酰脲作为目标物的方法进行了对比,发现方法样品用量及萃取剂用量均较少,且所需前处理时间较短,有机溶剂消耗也较少,为苯甲酰脲类农药的检测提供了更快速、绿色的选择。为评估所开发方法的实际样品适用性,将其应用于3种河水样品中4种苯甲酰脲类杀虫剂的分析检测,所得回收率及RSD分别为69.5%~99.4%和0.2%~9.5%,表明此方法在实际样品中同样具有较高的准确性和精密度。

## Abstract (en)

Pesticides are used in the agricultural production process to ensure the yield and quality of agricultural products. However, in recent years, environmental pollution issues caused by pesticide residues have sparked widespread concern in society. It is important to develop convenient and efficient approaches to detect and monitor pesticide residues. In this study, targeting benzoylurea insecticides (BUs), polyamidoamine dendrimer-functionalized silica nanocomposite with polydopamine coating (SiO_2_-PAMAM-PDA) was designed and successfully synthesized. First, monodisperse silica nanoparticles were prepared by the hydrolysis of tetraethyl orthosilicate (TEOS) in mixed solution of ethanol, water and ammonia. The silane coupling agent (3-aminopropyl)triethoxysilane was then employed to introduce amino groups into the silica. Silica with the zeroth generation of polyamidoamine (PAMAM) modification (SiO_2_-PAMAM-G0) was obtained through Michael addition reaction of methyl acrylate. Ethylenediamine was added to polymerize with methyl acrylate using an amidation reaction to form the first-generation PAMAM (SiO_2_-PAMAM-G1). Finally, by polymerizing dopamine under alkaline conditions (pH=8.5), the SiO_2_-PAMAM-G1 was coated with PDA. Thus, the final product named SiO_2_-PAMAM-PDA was obtained. The composite was characterized using a transmission electron microscope (TEM) and an increase in surface roughness indicated the successful grafting of PDA coating. Dopamine structure contains abundant benzene rings and amino and hydroxyl groups. It could bind with BUs through multiple secondary interactions, such as hydrogen bond and *π-π* stacking interaction. Therefore, the introduction of PDA could effectively enhance the affinity of the material toward benzoylurea insecticides. The prepared nanocomposites were used as sorbents in a dispersive micro solid-phase extraction approach (D-μ-SPE). The established approach was employed to extract and enrich the BUs in water samples before high-performance liquid chromatography (HPLC) analysis. Diflubenzuron, triflumuron, hexaflumuron, and teflubenzuron were chosen as target analytes. The following was a typical D-μ-SPE procedure. The prepared adsorbents measuring 40 mg were first dispersed in an 8-mL sample solution containing 150 g/L NaCl. The dispersion was assisted by 120-s vortexing to ensure full contact between the SiO_2_-PAMAM-PDA and the targets. Next, the adsorbents were separated from the liquid phase by 4-min centrifugation (5000 r/min). Thereafter, the adsorbed benzoylureas were eluted using 1 mL acetonitrile as desorption solvent by 120-s vortexing. Separated by centrifugation, the eluate was dried under a mild nitrogen stream. The solid remains were redissolved in 0.1 mL of acetonitrile, filtered by filter membrane (0.22 μm), and then analyzed by HPLC. The experimental conditions in the D-μ-SPE process could have a great impact on the extraction efficiency. Experimental conditions were optimized using a single factor optimization approach to further enhance the extraction recoveries. The optimized conditions included adsorbent amount, extraction time, desorption solvent type, desorption solvent volume, desorption time, and NaCl addition amount. Under the optimal conditions, a linearity range of 10-500 μg/L and limits of detection (LODs, *S/N*=3) of 1.1-2.1 μg/L were obtained. The extraction recoveries and relative standard deviations (RSDs) of the four BUs were 82.8%-94.1% and 2.1%-8.0%, respectively. The established approach was compared with reported approaches targeting benzoylurea insecticides. It was discovered that this approach consumed less sample, material, organic solvent, and pretreatment time. It provided a more rapid and green choice for the determination of benzoylurea pesticides. To determine the applicability, the proposed approach was applied to analyze the four benzoylurea insecticides in three river water samples. The real water samples were pretreated using the developed approach ahead of instrumental analysis, and no benzoylurea pesticides residue was detected. Next, standard addition experiments were performed under three spiking levels, including 15, 50, and 200 μg/L. The established approach had good accuracy and feasibility with satisfactory recoveries (69.5%-99.4%) and RSDs (0.2%-9.5%).

苯甲酰脲类杀虫剂(BUs)以杀虫活性高和环境持久性低等优势被广泛应用于害虫的防治,其主要作用于昆虫的幼虫阶段,通过抑制或阻断几丁质的合成,使昆虫因无法蜕皮而死,从而达到杀虫的目的^[1,2]^。然而杀虫剂的不规范施用会导致其在土壤、水和植物中的残留积聚,会对生态环境造成不良影响;长期使用、接触苯甲酰脲类杀虫剂也会对人类健康构成潜在危害。个别苯甲酰脲类杀虫剂,如除虫脲和氟虫脲,已被归类为中等毒性物质^[3,4]^。因此,开发一种快速、简单、灵敏的分析方法对苯甲酰脲类杀虫剂残留进行监控十分必要。

基质复杂的样品往往难以直接分析,需要对其进行预处理,以达到选择性分离、去除干扰物质和富集分析物的目的,从而满足仪器分析的要求^[5]^。目前有许多前处理技术,包括固相萃取(SPE)、分散微固相萃取(D-μ-SPE)、固相微萃取(SPME)、搅拌棒吸附萃取(SBSE)以及磁性固相萃取(MSPE)等^[6⇓-8]^。其中,D-μ-SPE技术是在分散固相萃取(DSPE)的基础上进一步简化、小型化发展而来的技术,具有机溶剂消耗少、萃取效率高、吸附剂便于回收及可重复利用的优点。D-μ-SPE的具体操作流程为:加入微量吸附剂,采用涡旋、超声等方式辅助吸附剂在样品溶液中均匀分散,从而对目标分析物进行提取,再通过离心、过滤等手段将固体吸附剂与溶液分离,解吸附后进行仪器分析^[9]^。吸附剂的物理化学性质对D-μ-SPE的灵敏性和选择性起着决定性的作用,性能优良的吸附剂需具备较高的比表面积和吸附容量,以及良好的分散性和稳定性。目前,用于D-μ-SPE的吸附剂主要有二氧化硅、多壁碳纳米管、氧化石墨烯等纳米材料以及多种功能性成分的复合材料^[10,11]^。

树状分子由中心核、高度重复的支化单元,以及外围的官能团组成,均匀性、主客体潜力和丰富的内部孔隙等结构特性使树状分子成为一类理想的吸附材料^[12]^。聚酰胺-胺(PAMAM)是最常用的一类树状分子,其结构简单,易于合成,具有丰富的氨基结构,目前已经被广泛应用于重金属离子吸附、药物萃取、染料吸附以及全氟辛酸、溴化阻燃剂等有害环境污染物的去除等方面,而其在农药残留检测中的应用鲜有报道^[13⇓⇓⇓-17]^。海洋生物贻贝类所分泌的丝足蛋白中富含3,4-二羟基-1-苯丙氨酸(DOPA),这使其可以黏附在几乎任何材料的表面^[18]^。受此启发,分子内部同样含有大量邻苯二酚官能团的多巴胺(dopamine, DA)引起了人们的关注并作为新型涂料得以广泛应用^[19]^。多巴胺在弱碱性条件下自聚合得到的聚多巴胺(PDA),其具有可控的涂层厚度以及良好的稳定性,丰富的羧基和氨基也赋予了PDA一定的吸附能力^[20]^。在农药检测前处理领域,PDA常用于碳基材料、层状双氢氧化物(LDHs)、金属有机骨架(MOFs)等材料的功能化,以构建新型复合吸附剂^[21]^。Xiong等^[22]^制备了PDA修饰的磁性石墨烯,用于水样中三唑类杀菌剂的前处理,富集因子高达572~916; Du等^[23]^将PDA涂敷在磁性的Mg/Al LDHs表面制得了三层纳米复合材料,结合高效液相色谱可实现果汁样品中痕量有机磷类农药的高精度检测;Deng等^[24]^利用MIL-101型铁基MOFs对负载PDA的Fe_3_O_4_纳米颗粒进行修饰,所得磁性材料可快速从环境水样及蔬菜样品中提取磺酰脲类除草剂。目前PDA与树状分子的结合仍鲜有报道,本研究团队在前期工作中已开发了基于PAMAM的吸附剂用于农药的分散微固相萃取,取得了较好的效果,PDA的引入有望进一步提高材料的萃取能力。

本研究设计合成了聚多巴胺涂敷的聚酰胺-胺功能化二氧化硅(SiO_2_-PAMAM-PDA),并以之作为分散微固相萃取吸附材料,同时结合高效液相色谱-二极管阵列检测器(HPLC-DAD),建立了一种水样中苯甲酰脲类杀虫剂残留的检测方法,拓展了聚多巴胺在样品预处理领域的应用。

## 1 实验部分

### 1.1 仪器、试剂与材料

Agilent 1100高效液相色谱仪(美国Agilent公司); KQ3200DB数控超声波清洗器(昆山市超声仪器有限公司)。

实验所用的农药标准品(除虫脲(diflubenzuron)、杀铃脲(hexaflumuron)、氟铃脲(triflumuron)、氟苯脲(teflubenzuron),其结构式见图1)、3-氨基丙基乙氧基硅烷(APTES)、丙烯酸甲酯、盐酸多巴胺等购自上海阿拉丁试剂公司;甲醇、乙腈、丙酮、乙酸乙酯、乙醇、乙二胺、正硅酸四乙酯(TEOS)、氯化钠等购自北京国药集团化学试剂有限公司;三羟基氨基甲烷(Tris)购自北京偶合科技有限公司。甲醇、乙腈为色谱纯,其他试剂均为分析纯。

**图1:**
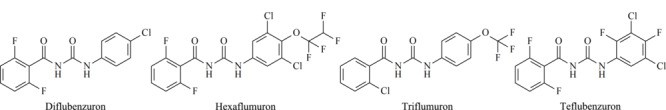
4种苯甲酰脲类目标物的结构式

### 1.2 色谱条件

色谱柱:Spursil C18柱(250 mm×4.6 mm, 5 μm);保护柱:Spursil C18柱(10 mm×2.1 mm, 5 μm);流动相A:水,流动相B:乙腈;柱温:30 ℃;流速:1 mL/min;进样量:10 μL;检测波长:220 nm。梯度洗脱程序:0~0.4 min, 70%B; 0.4~3 min, 70%B~52%B; 3~17 min, 52%B; 17~35 min, 52%B~70%B; 35~37 min, 70%B。

### 1.3 SiO_2_-PAMAM-PDA的制备

向3.6 mL水、10 mL氨水、100 mL乙醇混合体系中加入5.84 g TEOS,搅拌3 h。产物用50%乙醇水溶液洗涤多次,60 ℃真空干燥12 h,得到SiO_2_纳米颗粒。将2.5 g SiO_2_分散在75 mL乙醇中,超声30 min,加入20 mL三乙胺,在40 ℃下搅拌2 h。将1.5 mL APTES溶于20 mL乙醇中,缓慢加入上述体系,继续搅拌12 h。所得固体用丙酮洗涤3次后在60 ℃下干燥12 h,得到氨基化改性的二氧化硅纳米颗粒(SiO_2_-NH_2_)。

利用迈克尔加成和酰氨化反应向SiO_2_-NH_2_中引入树状分子。将1 g SiO_2_-NH_2_在超声辅助下分散于20 mL甲醇中。冰水浴条件下向溶液中缓慢加入20 mL丙烯酸甲酯,搅拌15 h。反应结束后,用甲醇洗涤产物多次,得到的产物记为SiO_2_-PAMAM-G0。将SiO_2_-PAMAM-G0分散于10 mL甲醇中,逐滴加入2 mL乙二胺,35 ℃下搅拌3 h。产物经甲醇洗涤后于60 ℃真空干燥12 h,得到第一代树状分子改性的二氧化硅纳米材料(SiO_2_-PAMAM-G1)。

将0.3 g SiO_2_-PAMAM-G1分散于70 mL乙醇-水(4∶3, v/v)中,超声30 min。加入0.3 g盐酸多巴胺,搅拌10 min后加入20 mL Tris溶液,调节体系pH值至8.5,反应24 h。产物经抽滤、洗涤、烘干后得到聚多巴胺涂敷的二氧化硅复合材料(SiO_2_-PAMAM-PDA)。

### 1.4 分散微固相萃取

向8 mL含有目标分析物且NaCl添加量为150 g/L的水样中加入40 mg SiO_2_-PAMAM-PDA,涡旋120 s,使吸附剂与溶液充分接触。之后以5000 r/min的速率离心4 min,移除上清液,将固体部分氮吹至干。将1 mL乙腈加入上述离心管中,涡旋120 s以对目标分析物进行解吸附,离心4 min将固体材料与解吸附溶剂分离,将上清液转移至新离心管中,氮吹至干。加入0.1 mL乙腈,涡旋30 s将残余物复溶,所得体系用滤膜(0.22 μm)过滤后进入HPLC检测。

## 2 结果与讨论

### 2.1 SiO_2_-PAMAM-PDA的制备优化及表征

在反应过程中,SiO_2_-PAMAM-G1和多巴胺的比例可能会影响多巴胺在材料表面的聚合程度,从而影响所得材料的萃取效果,因此按照1∶1、1∶3和1∶6的质量比合成了3批材料,并对其萃取效果进行了评价。如图2所示,在反应中投入更多的多巴胺并不能提升萃取效果,可能是由于SiO_2_-PAMAM-G1的表面位点有限,多余的多巴胺无法在材料表面很好地聚合。因此选用1∶1作为材料合成的投料比。同时,引入了多巴胺后,材料对于4种苯甲酰脲的萃取效果显著提升,可能由于多巴胺结构中含有丰富的苯环、氨基和羟基,可以通过*π-π*堆积、氢键等次级相互作用与同样富含苯环及强电负性原子的苯甲酰脲类化学物结合,增强了材料对目标物的亲和力。

**图2:**
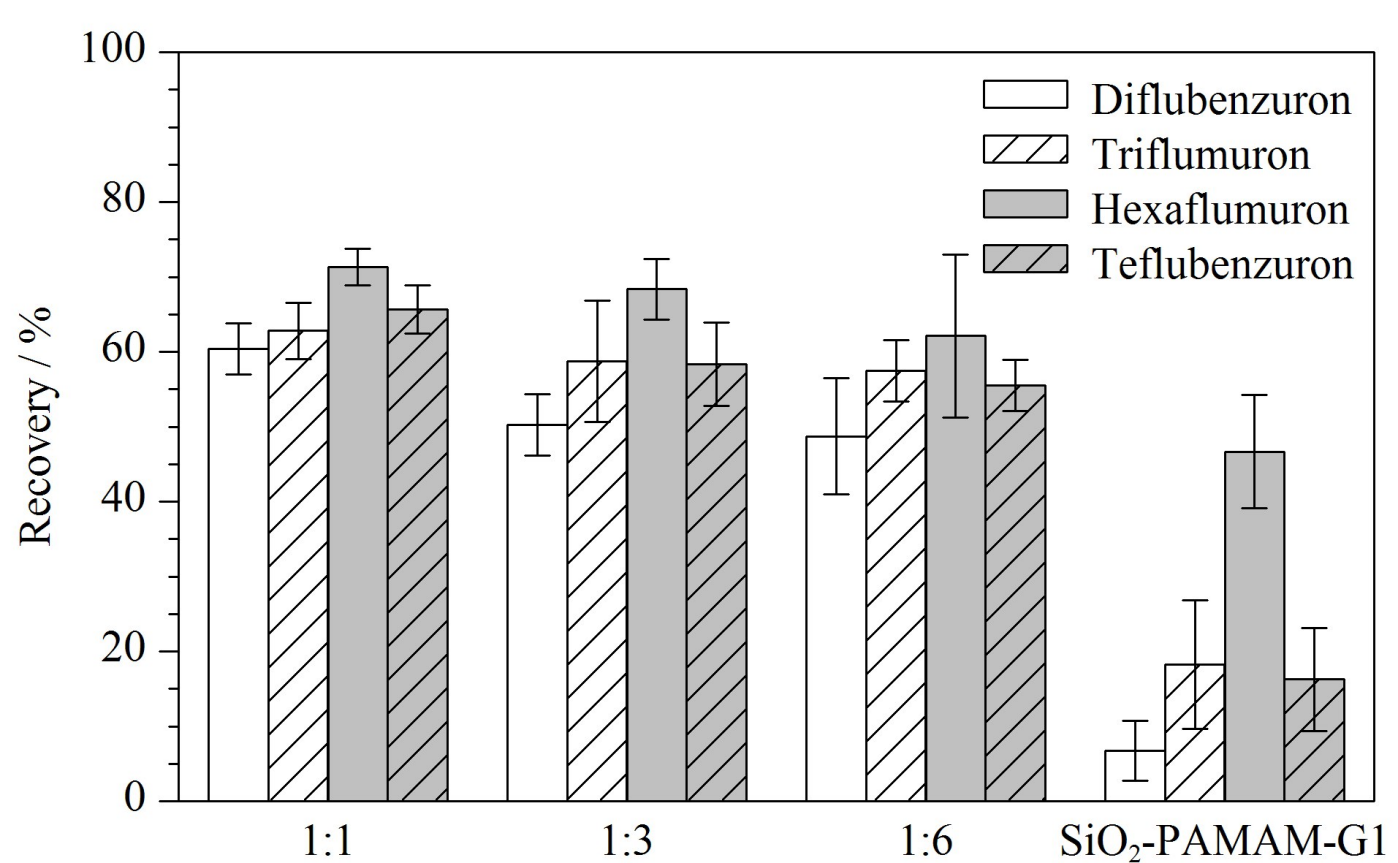
SiO_2_-PAMAM-G1和多巴胺的质量比对萃取效果的影响(*n*=3)

与SiO_2_-PAMAM-G1的透射电镜图相比(见图3a),SiO_2_-PAMAM-PDA的表面粗糙程度有所增加(见图3b和图3c),说明聚多巴胺成功涂敷在了SiO_2_-PAMAM-G1的表面。

**图3:**
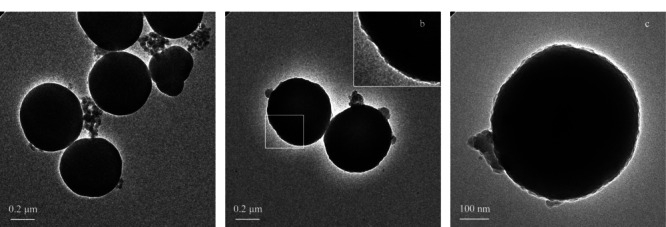
(a) SiO_2_-PAMAM-G1及(b, c) SiO_2_-PAMAM-PDA的透射电镜图像

### 2.2 萃取条件优化

#### 2.2.1 吸附剂质量

在萃取过程中,吸附剂的用量直接影响了萃取效果,吸附剂偏少会导致萃取效果不佳,过多则不仅会造成浪费,还有可能导致材料的团聚而不利于分散。如图4a所示,吸附剂用量达到40 mg时,4种目标物的萃取回收率均较高,进一步增加至50 mg时,回收率无明显变化。因此,选择40 mg吸附剂开展后续实验。

**图4:**
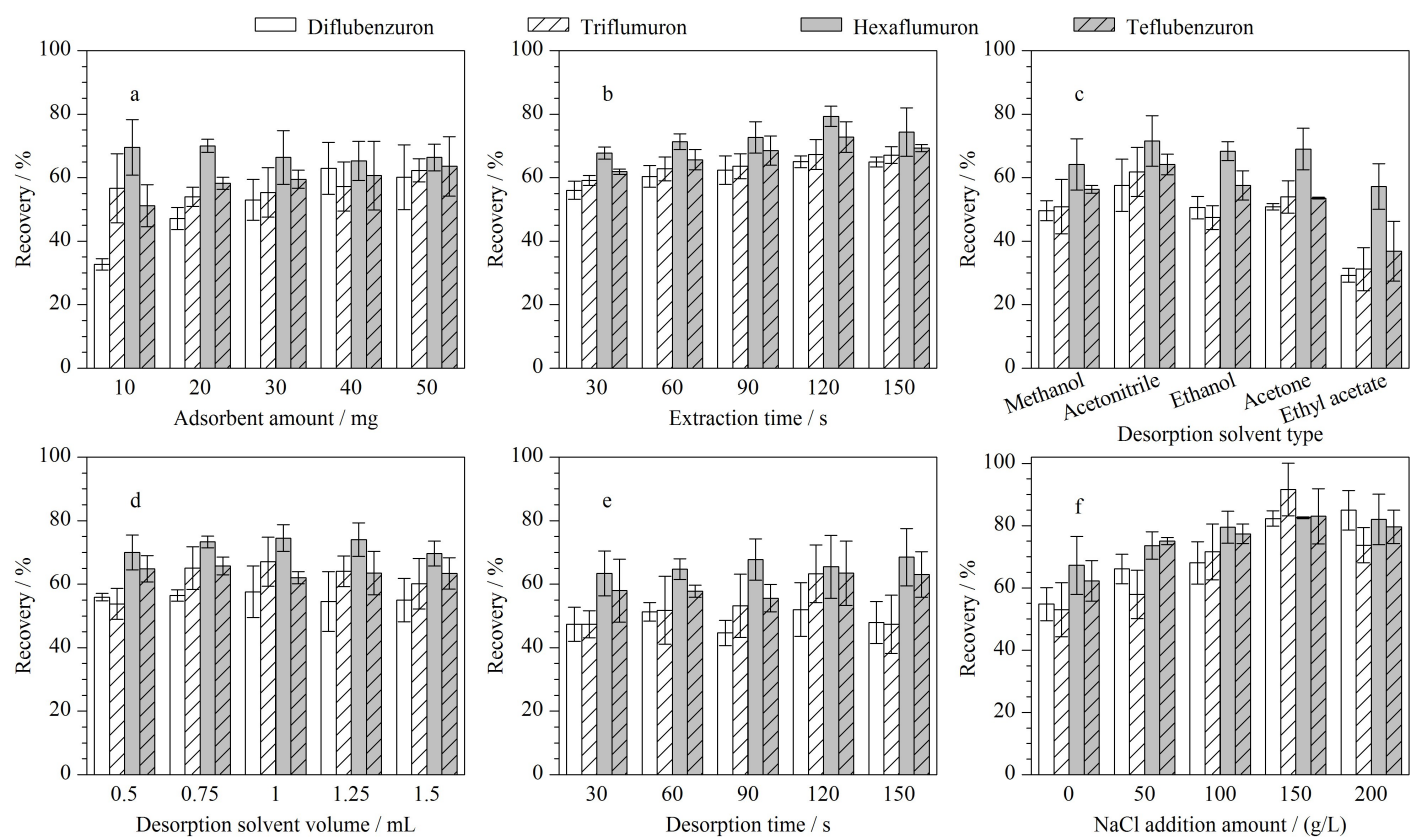
(a)吸附剂质量、(b)萃取时间、(c)解吸附剂类型、(d)解吸附剂体积、(e)解吸附时间及(f)NaCl添加量对萃取效率的影响(*n*=3)

#### 2.2.2 萃取时间

研究采用涡旋手段辅助吸附剂在样品溶液中的分散,充足的吸附时间保证了吸附剂与样品溶液的充分接触。探究了不同萃取时间(30、60、90、120、150 s)对萃取效率的影响(见图4b),可以看出,萃取回收率从30~120 s逐渐增加直至平衡,150 s时有所下降。因此,选择120 s作为最佳萃取时间。

#### 2.2.3 解吸附剂的种类

选择合适的解吸附剂将目标物从吸附剂上洗脱下来对萃取效果也十分重要,因此评估了甲醇、乙腈、乙醇、丙酮、乙酸乙酯共5种常用有机溶剂作为解吸附剂时的解吸附效果,结果如图4c所示。可以看出,使用乙腈作为解吸附剂时,回收率最高,表明乙腈可以有效破坏吸附剂与目标物的相互作用,因此后续实验均采用乙腈进行解吸附。

#### 2.2.4 解吸附剂体积

由图4d可以看出,除虫脲、杀铃脲、氟铃脲3种苯甲酰脲类杀虫剂在加入1 mL乙腈时萃取效率均较高,进一步增加体积回收率无明显变化。因此出于节约溶剂、减少污染的考虑,选择使用1 mL乙腈作为解吸附剂进行后续实验。

#### 2.2.5 解吸附时间

研究了解吸附时间对萃取回收率的影响,结果如图4e所示。可以看出,解吸附时间为120 s时,材料对4种农药的回收率均达到最佳;进一步延长解吸附时间,回收率反而下降,因此以120 s作为后续实验的解吸附时间。

#### 2.2.6 NaCl添加量

向样品中加入NaCl会影响样品溶液中离子组成、离子强度以及溶液的黏度,进而可能会影响萃取效率。研究了不同NaCl添加量(0、50、100、150、200 g/L)对萃取效果的影响。从图4f中可以看出,当NaCl的添加量为150 g/L时,回收率最高,继续增加回收率反而下降,这可能是因为过高的NaCl浓度使溶液黏度增大,不利于传质的进行。因此,后续选择150 g/L作为最佳NaCl的添加量。

### 2.3 方法验证

在单因素优化实验筛选出的最优条件下,以SiO_2_-PAMAM-PDA为萃取材料,4种苯甲酰脲类杀虫剂为目标物,以线性、准确性、灵敏度、重复性作为标准对所建立的方法进行了验证(见表1)。结果显示,此方法具有较宽的线性范围(10~500 μg/L),目标物色谱峰面积(*Y*)和质量浓度(*X*, ng/mL)呈现良好的线性关系,相关系数(*r*^2^)均≥0.9989。根据3倍和10倍信噪比(*S/N*)计算方法的检出限(LOD)和定量限(LOQ),分别为1.1~2.1 μg/L和3.7~7.0 μg/L。

**表1:** 4种苯甲酰脲类杀虫剂的回归方程、线性范围、相关系数、回收率、相对标准偏差、检出限和定量限(*n*=4)

Analyte	Regression equation	Linear range/(μg/L)	r^2^	LOD/(μg/L)	LOQ/(μg/L)	Recovery/%	RSD/%
Diflubenzuron	Y=0.6907X+7.2049	10-500	0.9994	1.1	3.7	89.6	2.7
Triflumuron	Y=0.7563X+1.0803	10-500	0.9998	2.1	7.0	94.1	2.1
Hexaflumuron	Y=1.4735X-12.686	10-500	0.9989	1.5	5.0	86.3	8.0
Teflubenzuron	Y=1.1315X-4.2734	10-500	0.9992	1.1	3.7	82.8	4.4

*Y*: chromatographic peak area; *X*: mass concentration, μg/L.

在50 μg/L的标准添加浓度下,4种苯甲酰脲类目标物的回收率为82.8%~94.1%,相对标准偏差值(RSD)为2.1%~8.0%(*n*=4),表明建立的方法具有较高的准确性和重复性。

### 2.4 与其他方法的比较

将本文所开发的方法与其他检测水中的苯甲酰脲的方法进行了比较,结果列在表2中。可以看出,此法由于检测仪器制约,LOD值较高,但所需样品量以及吸附剂用量均少于其他方法,且耗时也更短。表明所开发的方法较为绿色高效,具备实际应用的潜力,与更高灵敏度的分析仪器联用可以达到更低的检出限。

**表2:** 所建方法与其他方法的比较

Method	Sample volume/mL	Time/min	Material	LOD/(μg/L)	Ref.
SPME-HPLC-UVD	10	69	181.6 mg TiO_2_	0.026-0.082	[25]
MSPE-HPLC-UVD	100	25	16 mg β-CDP@Fe_3_O_4_	0.02-0.05	[26]
SPE-HPLC-UVD	50	51.25	100 mg TiO_2_	0.062-0.212	[27]
MMF-SPME-HPLC-DAD	20	85	-	0.026-0.075	[28]
ISFME-D-μ-SPE-HPLC-DAD	8	5	15 μL [HMIM]Cl+235 μL LiNTf_2_+	0.67-1.46	[29]
			3 mg Fe_3_O_4_/nSiO_2_/mSiO_2_		
D-μ-SPE-HPLC-DAD	8	12	40 mg SiO_2_-PAMAM-PDA	1.1-2.1	this work

UVD: ultraviolet detector; MSPE: magnetic solid phase extraction; MMF: multiple monolithic fiber; DAD: diode array detector; ISFME-D-μ-SPE: in situ solvent formation dispersive microsolid-phase extraction; *β*-CDP@Fe_3_O_4_: *β*-cyclodextrin polymer@Fe_3_O_4_; [HMIM]Cl: 1-hexyl-3-methylimidazolium chloride; LiNTf_2_: lithium bis[(trifluoromethane)sulfonyl] imide; nSiO_2_: nonporous silica; mSiO_2_: mesoporous silica.

### 2.5 实际样品的测定

选取北京市内三段河水(永定河、林河及潮白河)作为实际样品,实验前经0.45 μm滤膜过滤以除去悬浮杂质。为了验证本研究所提出的方法在实际水样中的适用性,在最佳实验条件下,对上述3个实际水样中的苯甲酰脲类杀虫剂残留进行了检测。结果显示(见表3),在3种加标水平下(15、50、200 μg/L), 4种苯甲酰脲类杀虫剂的回收率为69.5%~99.4%, RSD值在0.2%~9.5%之间;空白及加标实际水样经所建立的方法处理后所得色谱图如图5所示,4种苯甲酰脲类杀虫剂的出峰均未受到基质干扰。上述结果表明此法可用于实际水样中苯甲酰脲类杀虫剂的检测。

**表3:** 实际水样中4种苯甲酰脲类杀虫剂的测定结果、标准添加回收率及相对标准偏差(*n*=3)

Analyte	Spiked/(μg/L)	Recoveries, RSDs/%		
		Yongding He	Lin He	Chaobai He
Diflubenzuron	0	N.D.	N.D.	N.D.
	15	74.1, 4.3	73.0, 6.9	82.3, 4.6
	50	77.5, 2.9	78.4, 4.0	81.6, 3.5
	200	74.5, 2.7	75.8, 2.1	69.5, 4.0
Triflumuron	0	N.D.	N.D.	N.D.
	15	77.8, 9.5	99.4, 5.2	82.3, 0.9
	50	81.9, 1.5	96.3, 8.0	91.5, 2.0
	200	78.0, 5.0	85.9, 3.0	73.7, 4.3
Hexaflumuron	0	N.D.	N.D.	N.D.
	15	80.0, 7.2	88.1, 1.8	90.6, 6.0
	50	85.8, 4.8	95.1, 0.2	93.3, 6.9
	200	89.4, 3.0	92.6, 3.0	82.5, 2.2
Teflubenzuron	0	N.D.	N.D.	N.D.
	15	71.2, 1.5	88.0, 4.7	94.2, 5.3
	50	77.7, 2.3	82.4, 2.9	76.2, 0.8
	200	81.1, 3.0	86.7, 3.1	77.8, 1.6

N.D.: not detected.

**图5:**
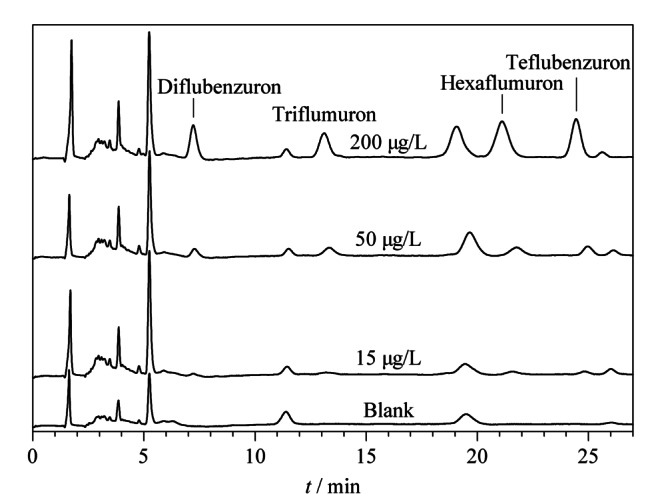
实际水样经所建立的方法处理后的高效液相色谱图

## 3 结论

本研究以聚多巴胺涂敷的聚酰胺-胺功能化的二氧化硅复合材料SiO_2_-PAMAM-PDA为吸附剂,通过分散微固相萃取法结合高效液相色谱对水样中的4种苯甲酰脲杀虫剂残留进行检测。结果显示,经聚多巴胺涂层修饰后材料的萃取性能得到了极大的提升,对4种杀虫剂均表现出良好的萃取效果。本文建立的方法拓宽了树状分子及多巴胺的应用范围,为水样中苯甲酰脲类杀虫剂残留的检测提供了更加绿色便捷的选择。
